# Substituent‐Dependent Structural Trends in Thermal Condensation of Silanetriols and Disiloxanetetraols

**DOI:** 10.1002/chem.71242

**Published:** 2026-06-12

**Authors:** Jan‐Falk Kannengießer, Svenja Pohl, Bernd Morgenstern, Oliver Janka, Guido Kickelbick

**Affiliations:** ^1^ Inorganic Solid‐State Chemistry Saarland University Saarbrücken Germany

**Keywords:** disiloxanetetraols, ladder‐type structures, organosilantriols, solid state condensation, thermal analysis

## Abstract

Oligo‐ and polysilsesquioxanes are typically synthesized via wet‐chemical routes employing acids or bases as catalyst. In this study we report a catalyst‐ and solvent‐free thermal condensation approach for the preparation of oligosilsesquioxanes from arylsilanetriols and their corresponding disiloxanetetraols. Three substituents, namely phenyl (Ph), 1‐napthyl (1Np), and 2‐naphthyl (2Np) were investigated along with their condensed products. Structural analysis revealed that the Ph and 1Np systems form ladder‐like oligosilsesquioxane architectures, whereas the 2Np system exhibits a markedly different structure, suggesting a layered arrangement of planar rings, as supported by FT‐IR and PXRD. Comparison of the silanetriols and disiloxantetraols as precursors indicated only minor differences, with crystal structures largely preserved after solid‐state condensation reactions. The thermal process achieved a higher degree of condensation than conventional wet‐chemical methods and enabled access to structurally distinct products without the need for solvents or catalysts, highlighting its potential for sustainable synthesis of novel polysilsesquioxane materials.

## Graphical abstract

We report a catalyst‐ and solvent‐free thermal condensation route to oligosilsesquioxanes from arylsilanetriols and disiloxanetetraols. Phenyl and 1‐naphthyl systems form ladder‐like structures, while 2‐naphthyl systems yields layered architectures. The condensation process preserves the crystal structures and achieves higher condensation than wet‐chemical methods, enabling sustainable access to distinct polysilsesquioxanes.

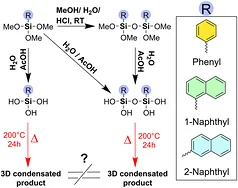

## 1 Introduction

Silanols are key intermediates in polycondensation reactions, such as the sol‐gel process [1] and the synthesis of polysiloxanes, typically formed during the hydrolysis of alkoxy‐ or chlorosilanes. Structurally they are silicon analogues of alcohols (C─OH), containing at least one Si─OH group. Despite this similarity, their chemical behavior differs markedly from that of carbon‐based alcohols. For example, geminal silanols containing multiple hydroxy groups on a single silicon atom (silanediols, and silanetriols) can be isolated, whereas geminal diols of carbon generally eliminate water to form carbonyl compounds, as described by Erlenmeyer's rule [2, 3, 4]. Although isolable, the stability of geminal derivatives decreases rapidly with an increasing number of silanol groups. This instability is primarily due to the lack of steric hindrance [1, 5, 6, 7]. Monosilanols with relatively small substituents on the silicon atom, for example, trimethylsilanol or triethylsilanol, can still be stable [1, 8], whereas silanetriols are only stable with sterically demanding substituents [1, 5, 9, 10]. As previously mentioned, another difference to carbon‐based alcohols is the high tendency of silanols to undergo condensation reactions [1, 5, 6, 11, 12]. Therefore, the synthesis of stable silanols, particularly silanetriols, can be challenging [5, 6, 13]. The known stable and isolable silanetriols often have sterically demanding substituents [14, 15, 16, 17], such as phenylsilanetriol [14, 18] and *tert*‐butyl silanetriol [17], which are probably the best known examples in literature. The relatively small number of single crystal X‐ray structures indicates the low stability and difficult isolability of silanetriols [19]. Cyclohexylsilanetriol is one of the few examples and again reveals that larger organic substituents are required for a high stability [16].

Silanetriols exhibit remarkable chemical versatility and have attracted attention for a variety of applications, which include their use as surface modification agents [9, 10, 11, 20, 21, 22], and as precursors for the synthesis of structurally complex compounds and polymers. Of particular relevance is their role in the formation of fully and incompletely condensed polyhedral oligosilsesquioxanes (POSS) [11, 23]. While silanediols have traditionally been employed in the polycondensation reaction of siloxanes, silanetriols remain underexplored in this context [24, 25, 26, 27, 28, 29]. Only the condensation of phenylsilanetriol in solution has been partially investigated to date [30]. Nevertheless, their trifunctional nature renders them promising precursors for crosslinked materials [9, 13].

Disiloxanetetraols are the monocondensed dimers of silanetriols. Examples are 1,3‐diphenyldisiloxane‐1,1,3,3‐tetraol [7, 31] and 1,3‐di‐(1‐naphthyl)disiloxane‐1,1,3,3‐tetraol [32]. Despite their structural simplicity, disiloxanetetraols have received limited attention in the literature, with only a few studies addressing their synthesis or characterization [7, 31, 32, 33, 34]. Consequently, no established applications for this class of compounds currently exist. Nevertheless, their potential as building blocks for the construction of well‐defined silsesquioxane architectures, such as ladder‐type or polyhedral oligomeric silsesquioxane (POSS) structures, appears promising and warrants further investigation [7, 34, 35].

The pronounced combination of Lewis acidity and basicity inherent to silanol groups enables the formation of exceptionally strong hydrogen bonds, particularly between adjacent silanol moieties (intra‐ or intermolecular) [1, 5]. These interactions are particularly prominent in the solid state [1, 11, 36, 37, 38]. Previous studies have shown that hydrogen bonds enable thermal condensation in solids and that structural elements of the original (crystal) structure are retained in the condensation product after this reaction [38]. However, these studies have predominantly focused on silanediols, which differ markedly from silanetriols in both molecular structure and the nature of their condensation products [5, 18].

The thermal condensation of silanols exhibiting extended hydrogen‐bonding networks in the liquid or solid‐state provides a solvent‐free and thus environmentally favorable alternative for the synthesis of polysiloxanes and related condensation products, which are typically produced in solution [23, 28, 29, 39]. This approach not only reduces the ecological footprint but also enables access to structural motifs that are inaccessible via conventional solvent‐based methods. The preservation of partial structural order during thermal treatment—absent in solution or molten compounds—leads to distinct condensation pathways and products [40, 41, 42, 43]. When applied to organosilanetriol precursors, this method is expected to yield fully condensed silsesquioxanes (SQs), a versatile class of organosilicon hybrid materials with the general formula (RSiO_1.5_)_n_ [44, 45]. SQs exhibit diverse architectures, including cage‐like, ladder‐type, and random network structures [44, 46, 47, 48]. Among these, ladder‐type polysilsesquioxanes (PSQs) are of particular scientific and technological relevance, owing to their well‐defined molecular arrangement, high thermal and chemical stability [45, 47], as well as their intrinsic rigidity and anisotropy [45]. Their structural features are typically characterized by distinct signatures observed in ^2^ ^9^Si solid‐state NMR and infrared spectra, which provide insights into the specific Si─O─Si bonding environments [45, 47].

**SCHEME 1:**
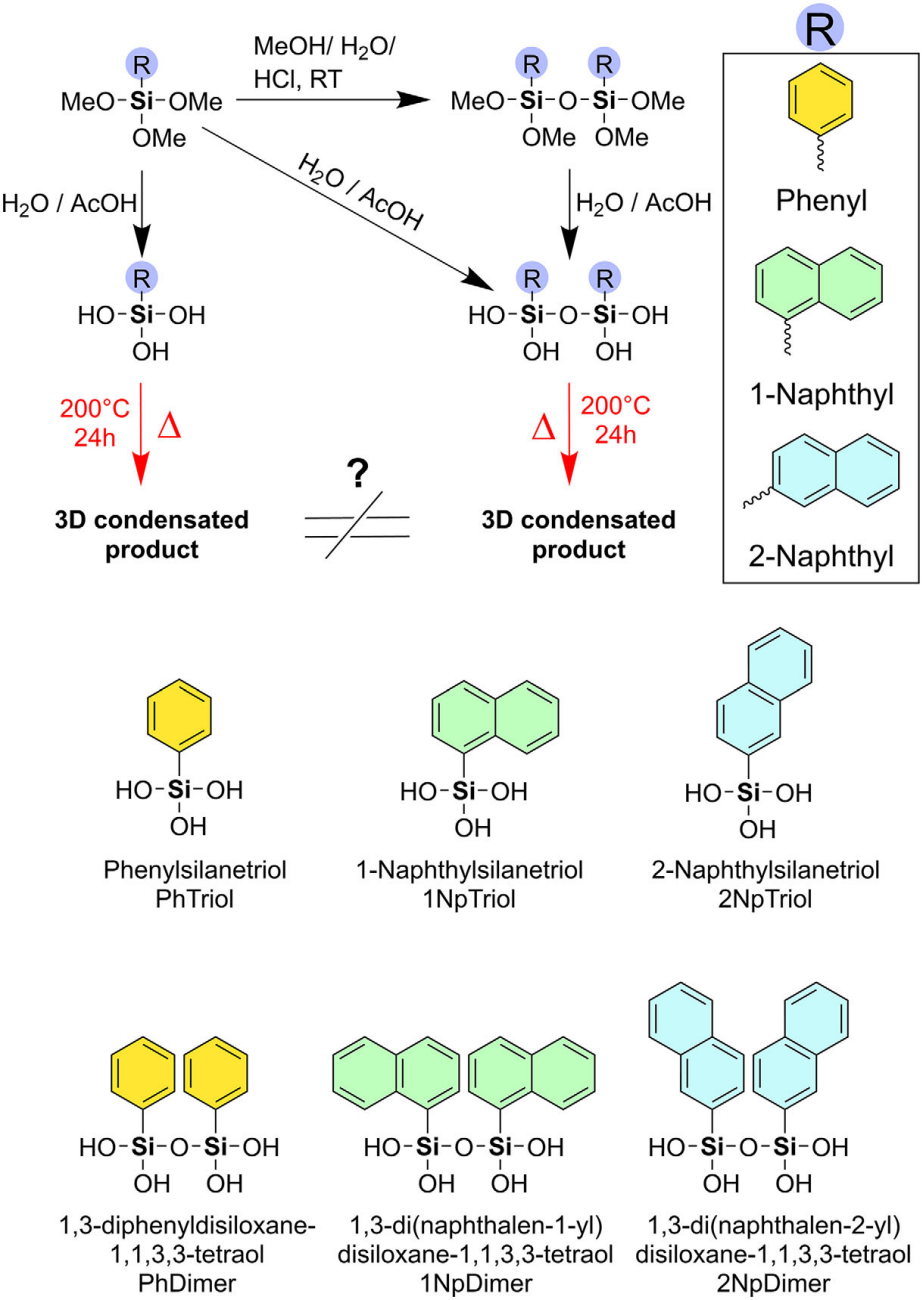
Synthesis scheme of the compounds investigated, including theoretical thermal condensation (red).

This study aimed to investigate the thermal condensation behavior of organosilanetriols by systematically comparing the reactivity of monomeric and dimeric species. In particular, structurally similar aryl substituents, such as phenyl, 1‐naphthyl, and 2‐naphthyl, were examined to gain mechanistic insights into the condensation process of silanetriols (Scheme 1). For each aryl system, both the corresponding silanetriol and its dimeric disiloxanetetraol were synthesized and subsequently subjected to thermal condensation under identical conditions. The resulting products were analyzed and compared with their precursors and with each other using a range of characterization techniques, including X‐ray diffraction (XRD), nuclear magnetic resonance (NMR), Fourier‐transform infrared spectroscopy (FT‐IR), and thermal analysis. The central focus of this investigation was to assess whether the condensation pathways differ between monomeric and dimeric precursors, potentially leading to distinct structural motifs. For example, silanetriols may favor the formation of six‐membered siloxane rings, whereas disiloxanetetraols, due to their connectivity, could form eight‐membered rings but are structurally incapable of forming six‐membered ones. In addition, the difference between our approach using silanols as precursors with thermal, solvent‐free condensation and the established methods, in which alkoxysilanes are used as precursors and the reaction takes place in solution to form so‐called melting gels [48, 49, 50, 51, 52] should also be investigated. We expect that the use of silanol precursors instead of alkoxysilanes results in differences in the reaction control and increased condensation. In addition, as precursors, silanols prevent unhydrolyzed alkoxy groups from entering the final products, which can have a significant impact on the properties due to the importance of hydrogen‐bonding patterns in the molten gel state. The reason for this is that unhydrolyzed alkoxy groups can act as hydrogen‐bond acceptors but not as donors.

## 2 Results and Discussion

The present study focused exclusively on aryl systems (phenyl, 1‐naphthyl, 2‐naphthyl) because of the known high stability of silanols formed in presence of these groups. Organosilanetriols and their corresponding dimers were synthesized via mild hydrolysis of trialkoxysilanes and disiloxane alkoxy derivatives using acetic acid as the hydrolyzing agent (Scheme 1) [37, 38]. The disiloxane alkoxy precursors were obtained by partial condensation of the trialkoxysilanes employing HCl (Scheme 1) [37, 38]. Each synthesis required careful optimization of reaction parameters including acid concentration, temperature, and reaction time, to ensure the isolation of the desired silanol species. Excessive acidity, elevated temperatures, or prolonged reaction times led to premature condensation of the silanols. In some cases, the target silanol precipitated directly from the reaction mixture, while in others, rapid termination and immediate further processing of the reaction was necessary to prevent further condensation and to enable the isolation of the molecular silanol.

For thermal condensation (Scheme 1), the corresponding silanols were placed in snap‐cap vials and heated at 200 °C for 24 h. The condensation temperature was selected taking into account the DSC data to ensure complete condensation of all compounds under consideration. This meant that the condensation temperature was set above the melting points of all compounds.

### 2.1 NMR Spectroscopy

NMR spectroscopy was employed to verify the successful synthesis of silanetriols and disiloxanetetraols in solution and to investigate the structure of condensed products by solid‐state NMR. It further enabled the identification of smaller molecules, such as cyclic side products formed during condensation. These species were extracted from the solid products and characterized by NMR. Additionally, NMR was used to detect volatile molecules cleaved from the crosslinked backbone at elevated temperatures.

^1^H and ^29^Si NMR spectroscopy confirmed the successful hydrolysis of the alkoxysilanes. In particular, ^29^Si NMR allows identification of silanetriols and ‐tetraols [18, 32, 34, 53]. The structural similarity of the organic substituents provides insight into electronic effects influencing the silanol group.

**FIGURE 1:**
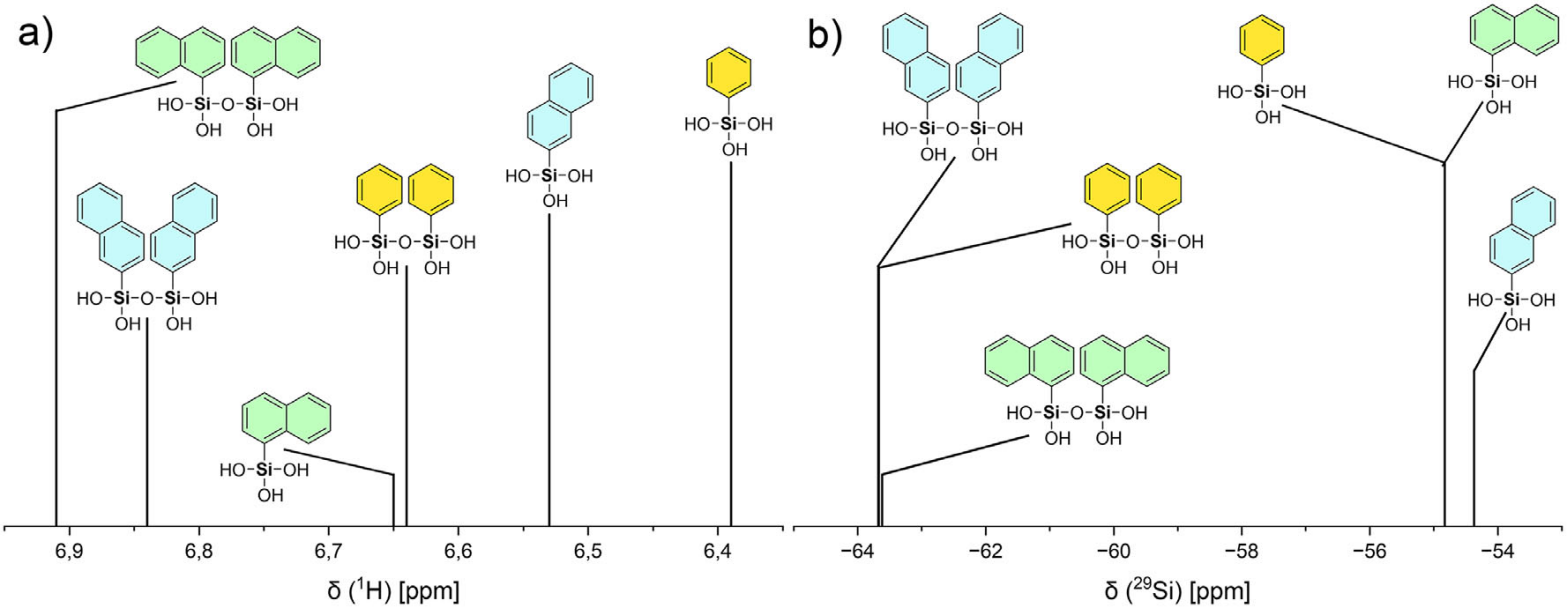
Summary of the ^1^H NMR results of the OH group (a) and ^29^Si NMR (b) results of the compounds used. All spectra were measured in DMSO‐d_6_.

In the ^1^H NMR spectra, dimers exhibit higher chemical shifts of the silanol proton than monomers (Figure 1a), with downfield shifts of 0.25–0.30 ppm relative to silanetriols, indicating increased acidity [54]. Within both series, phenyl‐substituted compounds are most shielded, while 1‐naphthyl derivatives are most deshielded due to aromatic anisotropy [55, 56]. The additional aromatic ring in 1‐naphthyl enhances this effect, whereas in 2‐naphthyl its greater distance reduces deshielding. Phenyl substituents show the weakest effect due to lower anisotropy.

In contrast, ^29^Si NMR spectra show significantly lower chemical shifts for dimers (Δ*δ* of approx. 9 ppm) compared to silanetriols (Figure 1b). In Si─O‐containing compounds, chemical shifts are mainly governed by paramagnetic contributions, which decrease with increasing condensation [57].

### 2.2 Solid‐State NMR Measurements

**FIGURE 2:**
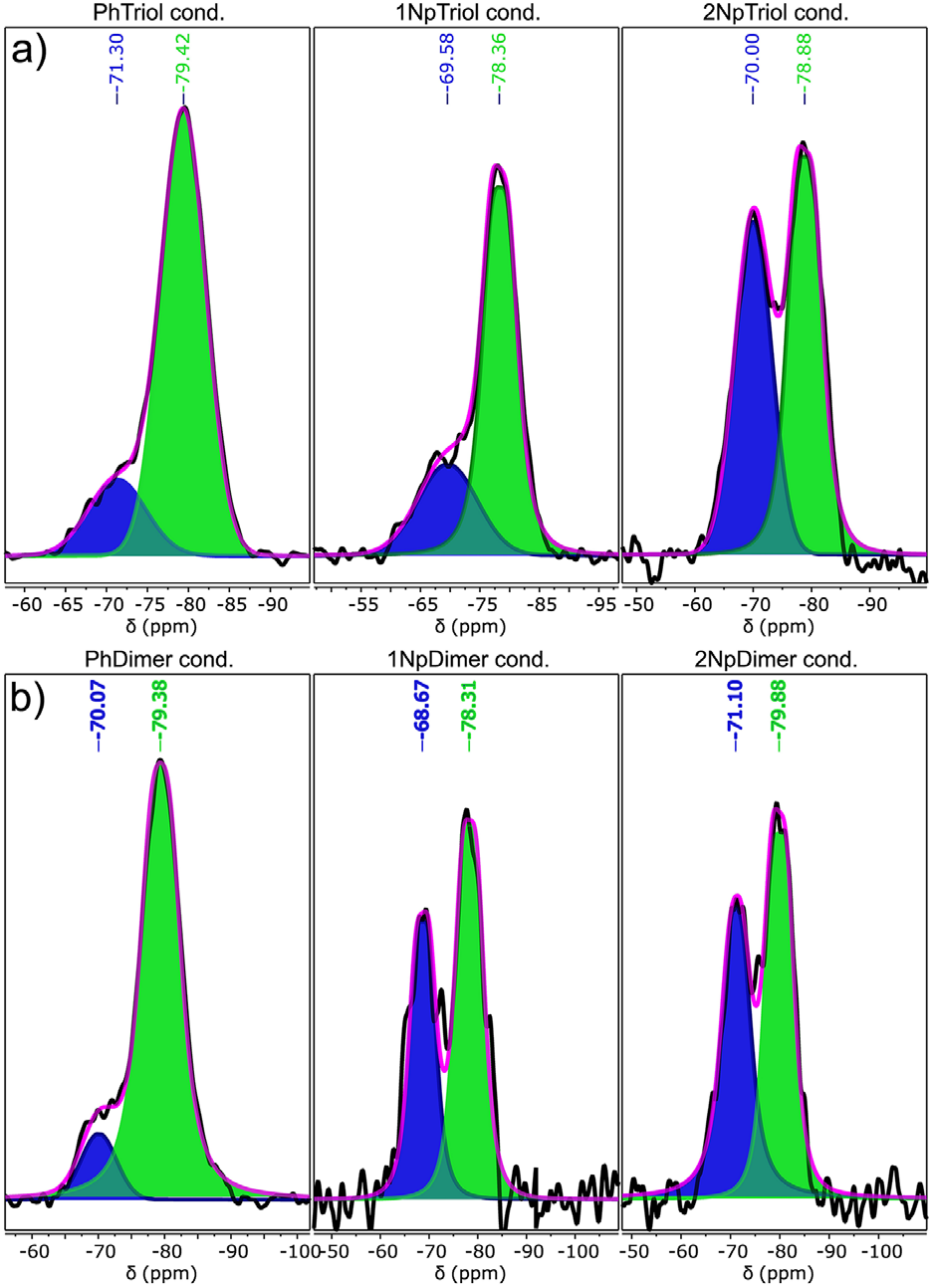
Deconvoluted ^29^Si SP‐MAS NMR spectra of the condensed silanetriols (a) and the condensed disiloxantetraols (b), with T^2^ units (blue) and T^3^ units (green).

After thermal condensation, the condensed products were analyzed by solid‐state ^29^Si SP‐MAS NMR spectroscopy. Spectral deconvolution enabled assignment of signal contributions and quantification of silicon environments (Figure 2). All samples exhibited only T^2^ and T^3^ units, with no evidence of T^1^ species, consistent with their expected low frequency as terminal groups after extensive condensation. The degree of condensation (DC) was calculated from the T^2^/T^3^ ratio (Table 1) [48, 58].

**TABLE 1:** Percentage proportions of T^2^ and T^3^ units of condensed compounds, including degree of condensation (DC).

Compound	Ph Triol / Dimer	PhMG_cons_ [48]	1Np Triol / Dimer	1‐NpMG_cons_ [49]	2Np Triol / Dimer	2‐NpMG_cons_ [49]
T^2^ (%)	18 / 10	51	28 / 41	69	46 / 49	47
T^3^ (%)	82 / 90	49	72 / 59	24	54 / 51	48
DC (%)	94 / 97	83	91 / 86	72	85 / 84	81

Among the aryl substituents, the phenyl system shows the highest DC (94–97%), while 2‐naphthyl exhibits the lowest (84–85%); 1‐naphthyl is intermediate (86–91%). Compared to literature on trimethoxysilane precursors, the observed DC values are comparable or higher (PhMG_cons_: 83%; 1‐NpMG_cons_: 72%; 2‐NpMG_cons_: 81%) [48, 49]. This is attributed to prior hydrolysis, as silanol hydroxy groups are more reactive than alkoxy groups, promoting condensation. The highest DC values are also consistent with alkyl‐substituted silsesquioxane systems [44].

Precursor comparison shows that in the phenyl system, the dimer (97%) condenses more extensively than the triol (94%). In contrast, for both naphthyl systems, dimers exhibit lower DC than the corresponding triols (1Np: 86 vs. 91%; 2Np: 84 vs. 85%). The ^29^Si solid‐state NMR peak patterns differ markedly between triol and dimer in the 1‐naphthyl system, whereas phenyl and 2‐naphthyl systems show similar patterns (Figure 2). Accordingly, the precursor‐dependent DC variation is most pronounced for 1‐naphthyl and negligible for phenyl and 2‐naphthyl (Table 1).

During thermal condensation, in addition to undefined oligosiloxanes, smaller discrete species such as siloxane rings or silsesquioxane cages may form and become incorporated into the products. In bulk measurements, these species are difficult detect and to distinguish from the network structure. Therefore, their extractability from the final materials was investigated.

To probe soluble fractions, 50 mg of the condensation products were suspended in 1 mL acetone‐d_6_, shaken for 1 h, and centrifuged. The supernatant was immediately analyzed by ^1^H NMR spectroscopy. Only weak signals were observed in the aromatic region. Their broadness indicates undefined oligomeric siloxane species rather than discrete cyclic or cage‐like compounds.

Although trace amounts of soluble material are present, no spectroscopic evidence supports the formation of well‐defined siloxane rings or silsesquioxane cages under the applied conditions.

### 2.3 FT‐IR Spectroscopy

Silsesquioxane structures exhibit characteristic FT‐IR bands in the range of 950–1200 cm^−^^1^, associated with cages, rings, and ladder‐like motifs. Ladder structures are identified by bands at 1030–1050 (ѵ_ring‐sym._) and 1130–1150 cm^−^^1^ (ѵ_ring‐asym._) [46, 47, 48]. Higher‐frequency bands correspond to more symmetrical (cage‐like) frameworks, whereas lower‐frequency bands indicate less symmetrical ladder structures [46, 47, 48]. Bands at 1000–1025 cm^−1^ are assigned to strained cycles, while cage structures typically appear at 1100–1125 cm^−1^ [47].

**FIGURE 3:**
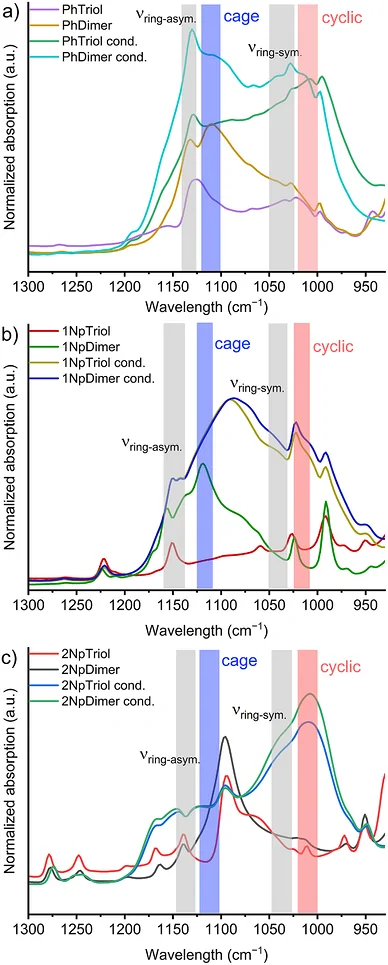
FT‐IR spectra of the silanetriols, dimers and condensed species of the phenyl system (a), the 1‐naphthyl system (b) and the 2‐naphthyl system (c).

In the phenyl system, uncondensed precursors differ mainly by a stronger band at 1115 cm^−1^ for PhDimer, attributed to the pre‐existing siloxane bond. Upon condensation, both ladder‐characteristic bands are clearly observed with comparable relative intensities (Figure 3a). The PhTriol‐derived product resembles literature data (phenyl melting gel) [48], whereas the PhDimer‐derived material differs significantly. A higher relative intensity of the low‐frequency band (1040 cm^−1^) in PhTriol indicates less symmetrical, ladder‐type structures, while the stronger high‐frequency band (1130 cm^−1^) in PhDimer suggests more symmetrical, cage‐like motifs. Cyclic bands at 1015 cm^−1^ are similar in both precursors.

In the 1‐naphthyl system, precursor differences are likewise limited to the 1115 cm^−1^ band. The condensed products show both ladder bands and an additional broad band at 1080 cm^−1^ (Figure 3b), with only minor differences between them. Both exhibit a cyclic band at 1015 cm^−1^, absent in the precursors.

In the 2‐naphthyl system, both precursors display a band at 1090 cm^−1^, which persists weakly after condensation and is attributed to the substituent. The condensed products are nearly identical (Figure 3c) but differ markedly from the other systems: the high‐frequency band at 1130 cm^−1^ is largely absent, while a dominant cyclic band at 1015 cm^−1^ with a shoulder at 1030 cm^−1^ is observed. This pattern indicates a disordered structure with strained ring‐like motifs [46, 47]. In contrast to literature reports (2‐naphthyl melting gel) [49], which show phenyl‐like spectra, the absence of high‐frequency bands here likely arises from differing synthesis conditions (thermal, solvent‐free vs. wet‐chemical, trialkoxysilanes).

### 2.4 Fluorescence Spectroscopy

The naphthyl‐substituted compounds and their condensed products exhibit pronounced fluorescence, enabling the investigation of aggregation via excimer emission. Solid samples were excited at 285 nm, corresponding to π‐π* transitions of the aromatic system [59]. To distinguish intra‐ from intermolecular excimers, dilution series in DMSO were performed: intermolecular excimer emission decreases with dilution, whereas intramolecular excimers remain largely unaffected, allowing structural interpretation, since intramolecular excimers indicate a partially ordered structure of the aryl substituents.

**FIGURE 4:**
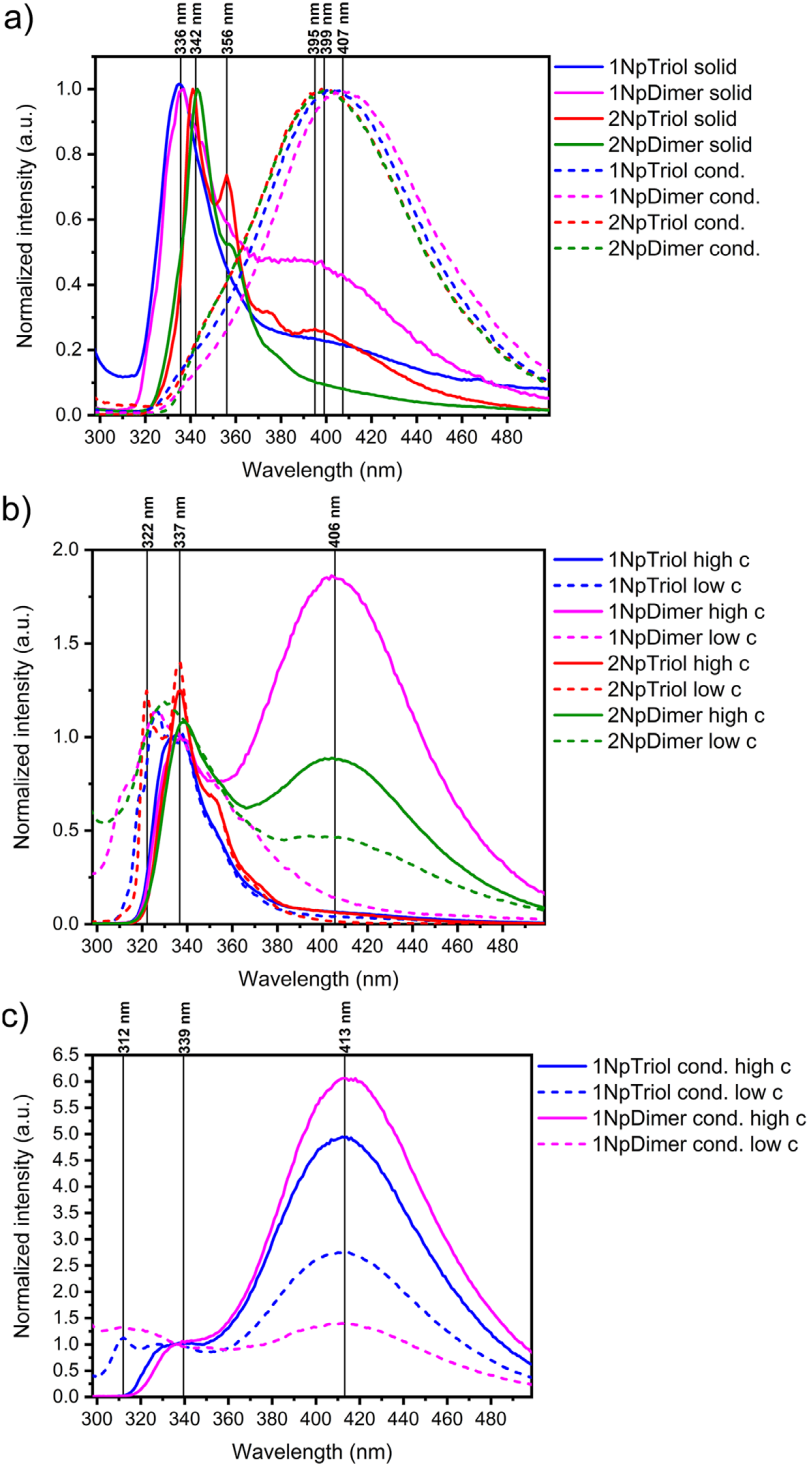
Fluorescence spectra of the compounds studied: spectra of the solid precursors and condensation products (a); dilution series of the precursors (b); dilution series of the soluble condensed species (c). The low concentrations (low c) varied between 6 × 10^−8^ and 1.2×10^−3^ g/L and high c between 0.6 and 5.3 g/L.

The molecular silanols show similar spectral features (Figure 4a). Monomer fluorescence appears at 330–360 nm, while excimer emission occurs at 390–410 nm [60]. Both silanetriols exhibit weak excimer emission in the solid state and none in solution. In contrast, dimeric species behave differently: 1NpDimer shows weak solid‐state but pronounced, concentration‐dependent excimer emission in solution, which disappears upon dilution, indicating intermolecular excimers. 2NpDimer shows no excimer emission in the solid state but exhibits partially concentration‐dependent emission in solution that persists at high dilution, suggesting both inter‐ and intramolecular excimers (Figure 4b).

The condensed species are dominated by excimer emission (390–410 nm) with minimal monomer contribution (330–360 nm), indicating reabsorption effects (Figure 4a). Due to limited solubility, dilution studies were only possible for the 1Np system (Figure 4c). All condensed samples show strong excimer emission in the solid state. In solution, both 1Np‐derived materials exhibit concentration‐dependent excimer fluorescence that persists at low concentrations, again indicating mixed inter‐ and intramolecular excimer formation. Notably, the 1NpTriol‐derived product shows significantly stronger excimer emission than the 1NpDimer‐derived analogue, suggesting structural differences between the condensation products.

Literature reports on 1Np‐ and 2Np‐functionalized melting gels [49] and their trimethoxysilane precursors describe pronounced solid‐state excimer fluorescence, whereas the silanetriols investigated here show only weak emission under comparable conditions. In solution, neither system exhibits excimer fluorescence.

Previously reported melting gels [49] displayed exclusively excimer emission, similar to the condensed species in this work. However, the consolidated melting gels described in the literature show a higher proportion of intramolecular excimers than the condensed species examined here. This difference points to structural variations between the literature systems and the materials investigated in this study.

### 2.5 X‐Ray Diffraction

**FIGURE 5:**
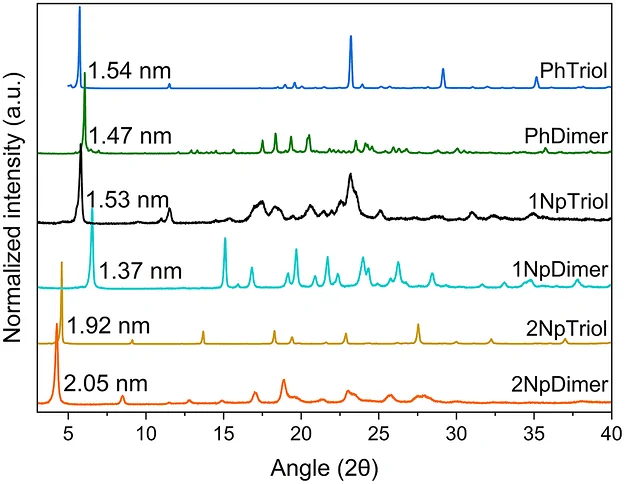
Powder diffractograms of the silanetriols and their dimers.

X‐ray diffraction proved to be a suitable method for elucidating the structural features of silanetriols, disiloxanetetraols, and their condensed species. The powder diffractograms of the precursors confirm their crystalline nature (Figure 5), showing intense low‐angle reflections used to calculate characteristic distances via Bragg's equation.

A trend can be observed in the phenyl derivatives, where the interlayer spacing decreases from 1.54 nm (PhTriol) to 1.47 nm (PhDimer), as well as in the 1‐naphthyl derivatives (1.53 nm to 1.37 nm). In contrast, the 2‐naphthyl system exhibits larger spacings overall, which increase upon dimer formation (1.92 nm to 2.05 nm).

Attempts to obtain single crystals of silanetriols and disiloxanetetraols were hindered by the pronounced condensation tendency of silanols, particularly for silanetriols. Consequently, crystal structures are available for only two compounds. While the structure of PhDimer has been previously reported [7, 31], that of 1NpDimer was so far derived only from powder data [32]. In this work, the single‐crystal X‐ray structure of 1NpDimer was successfully determined.

**FIGURE 6:**
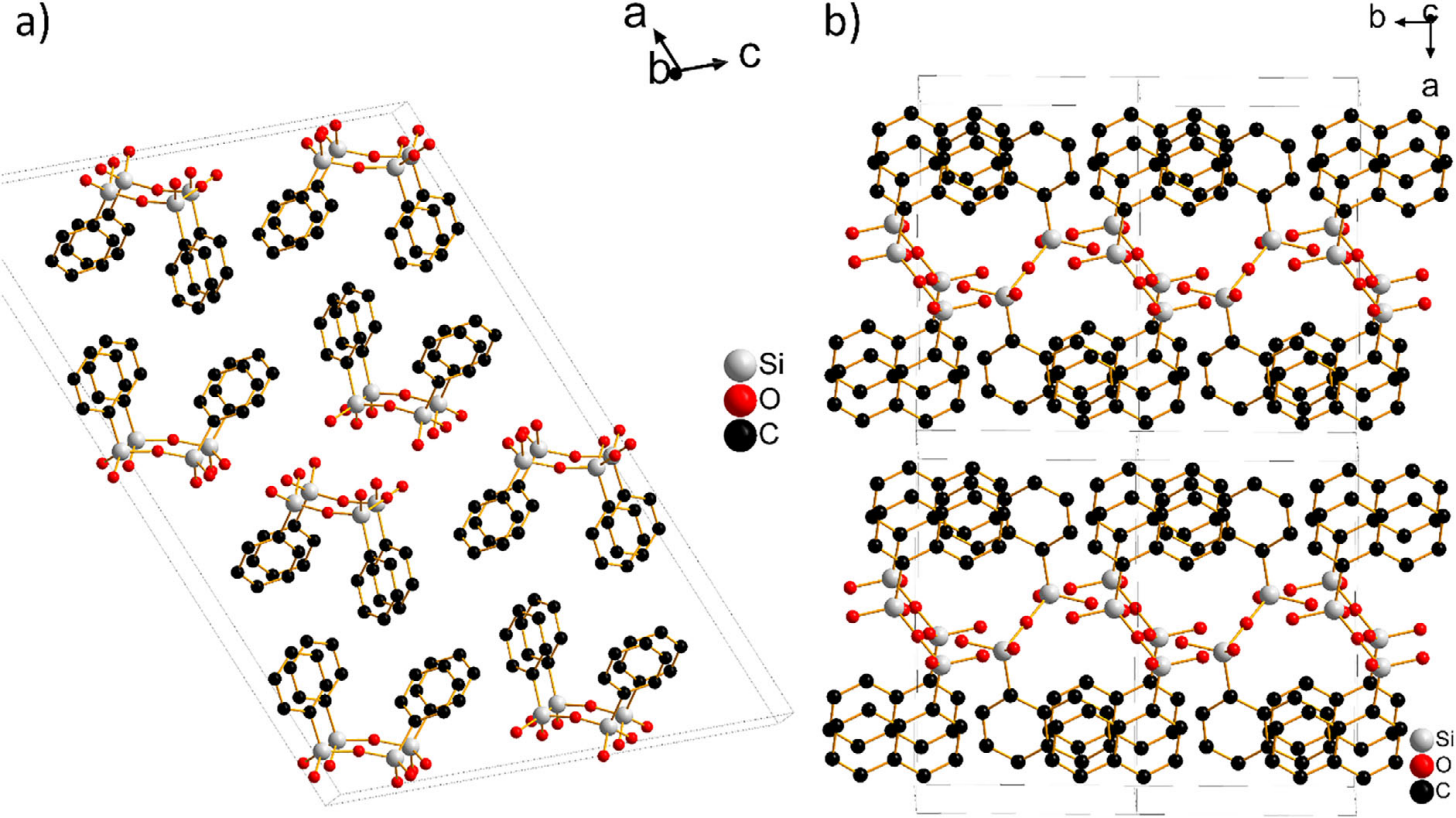
Crystal structures determined from single crystals from PhDimer (a) and 1NpDimer (b). Hydrogen atoms have been removed for clarity. The structure from PhDimer was taken from the literature [7, 31].

The experimental powder pattern of PhDimer does not match the simulated diffractogram from reported single‐crystal data (Figure 6a), likely due to polymorphism arising from synthesis‐dependent structural variations [7, 31]. In contrast, the simulated pattern based on the single‐crystal data of 1NpDimer (Figure 6b) agrees well with the experimental PXRD data [32]. The crystal structure of 1,3‐di‐(1‐naphthyl)disiloxane‐1,1,3,3‐tetraol (1NpDimer) revealed a layered arrangement in which the silanol groups lie in one plane and the aryl groups face each other. The silanol groups within the plane are linked to one another via hydrogen bonds. Four dimer molecules are located on one edge in the center of the unit cell and are aligned in the same direction. Exactly in the center of the unit cell is a fifth dimer molecule, which is rotated by 90° in the *bc* plane. This central dimer molecule is then connected to all outer dimer molecules via hydrogen bonds. The Si─O─Si bond angle of the dimers is 180°.

As mentioned earlier, both available single‐crystal structures (PhDimer, 1NpDimer) reveal layered arrangements, in which silanol groups form sheet‐like hydrogen‐bonded networks. The aligned aryl substituents define the interlayer spacing, corresponding to intense low‐angle reflections ((200) for PhDimer, (100) for 1NpDimer). As these planes are orthogonal to the hydrogen‐bonded silanol layers, the spacing does not originate within the silanol plane.

Although no single‐crystal data are available for silanetriols, their powder patterns suggest similar layered structures. Their crystal structures likely resemble those of the corresponding dimers, with hydrogen bonds connecting the condensation sites. This explains the similar interlayer distances of PhTriol (1.54 nm) and 1NpTriol (1.53 nm), reflecting comparable steric demand of phenyl and 1‐naphthyl substituents. In contrast, the bulkier 2‐naphthyl group leads to a larger spacing (1.92 nm, Figure 5).

**SCHEME 2:**
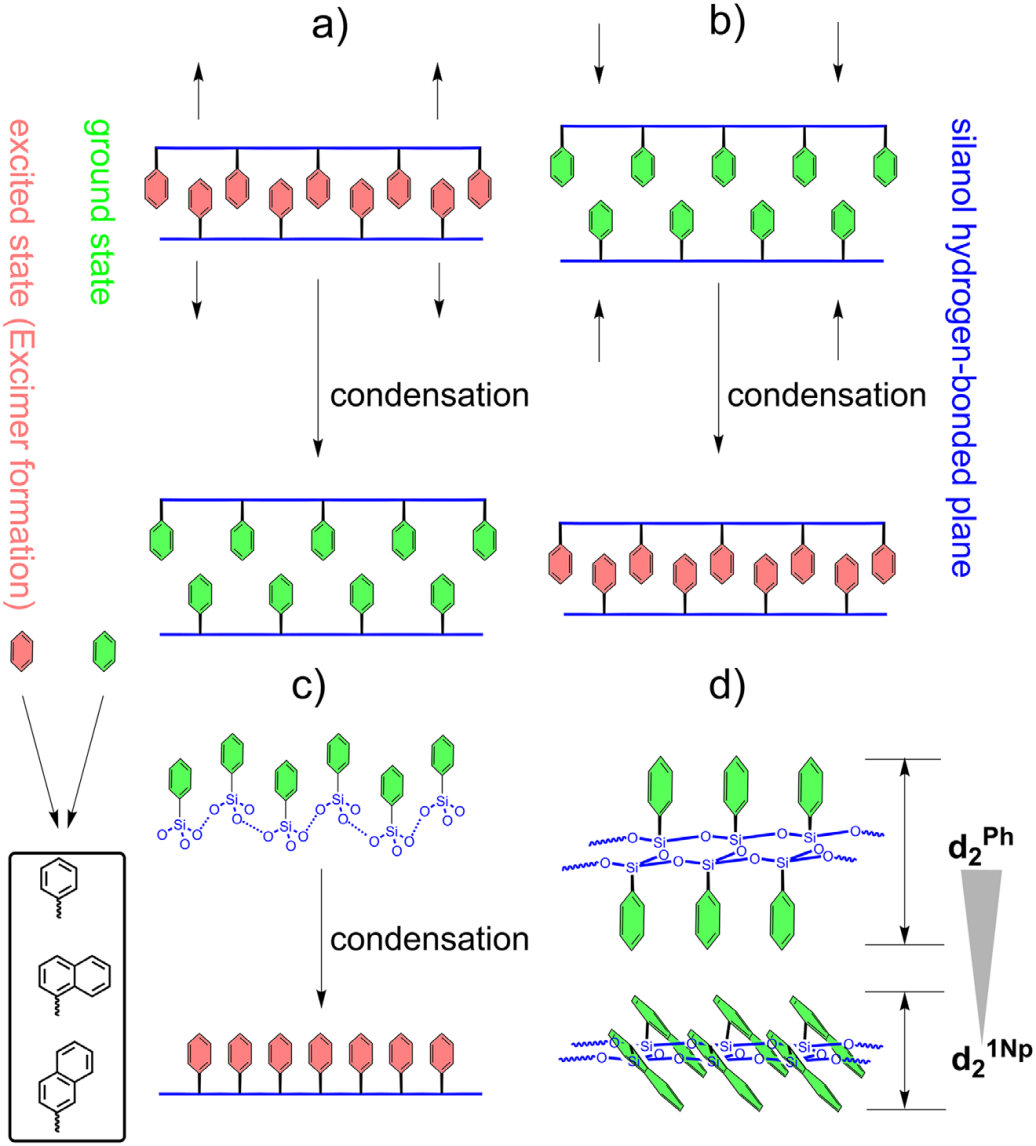
Schematic representation of the dilation (a) and contraction (b, c) of the planes, which weakens or strengthens excimer formation. Representation of the ladder structures with different orientations of the aryl groups, which changes the ladder thickness (d).

Condensation to disiloxanetetraols converts silanol groups into Si─O─Si linkages, increasing structural density but occurring within the hydrogen‐bond plane and thus not directly affecting interlayer spacing. The observed contraction therefore requires an additional structural adjustment, plausibly a rotation of the aryl substituents. In the phenyl and 1‐naphthyl systems, this facilitates interdigitation of aryl groups, reducing layer distance via interlayer sliding or improved packing (Scheme 2b,c), the latter also promoting excimer formation. In contrast, in the 2‐naphthyl system, condensation disrupts pre‐existing intercalation, leading to increased layer separation (Scheme 2a).

This interpretation is supported by fluorescence data (Figure 4): condensation of 1NpTriol enhances excimer emission, consistent with increased interpenetration, whereas 2NpTriol shows excimer fluorescence that disappears upon dimer formation, indicating loss of interlocking and increased spacing.

**FIGURE 7:**
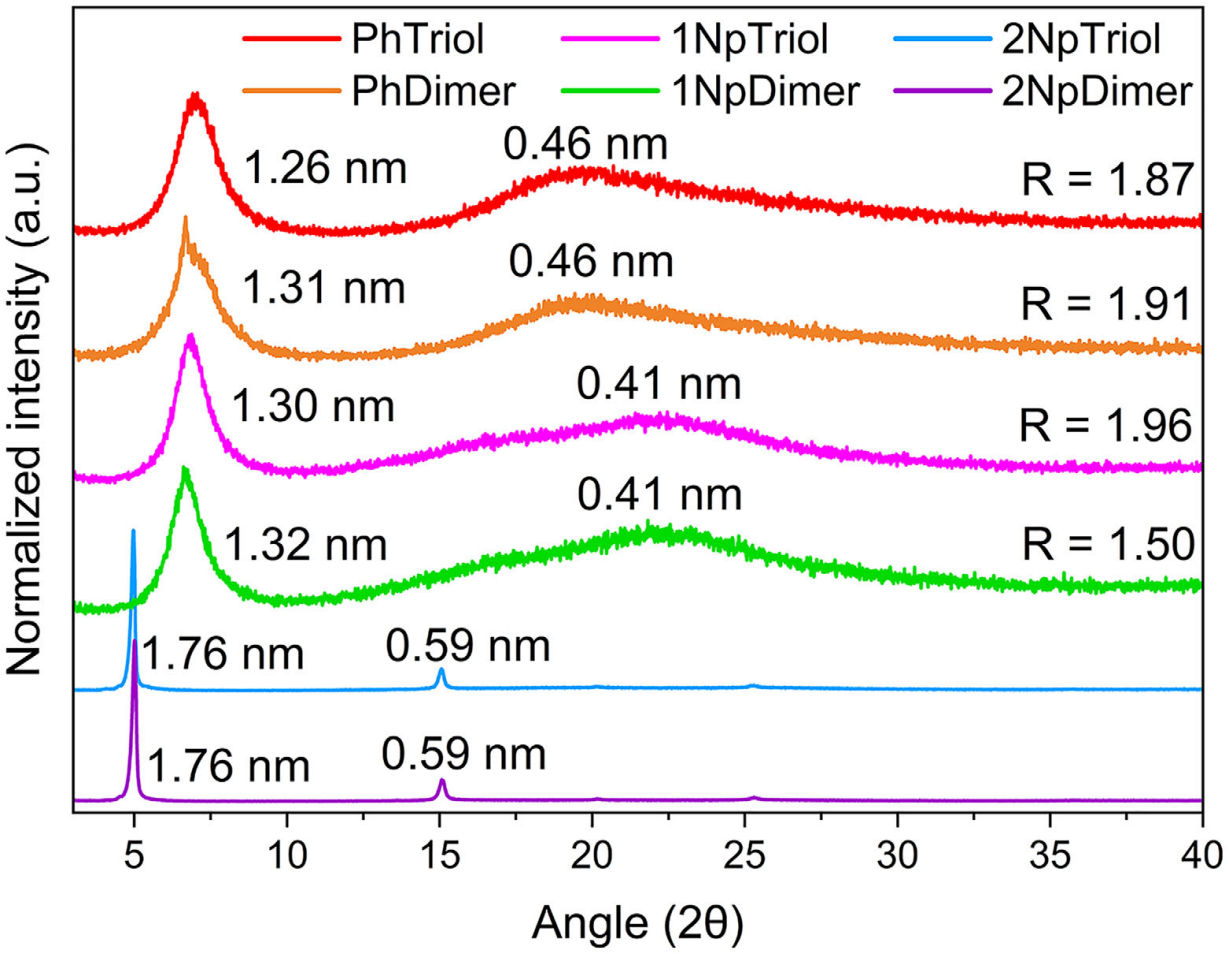
Powder diffractograms of the condensed species derived from the condensation of the silanetriols and their dimers.

For the condensed phenyl and 1‐naphthyl systems, diffraction patterns show the characteristic reflections of ladder‐type polysilsesquioxanes (Figure 7) [48, 50]. Two broad reflections around 7 and 20° 2*θ* correspond to chain‐to‐chain spacing (*d_1_*) and ladder thickness (*d_2_*) [35, 48, 50]. The intensity ratio (*R*) serves as a measure of ladder regularity [50]; values ranging from 1.50 to 1.96 (Figure 7) fall within the range reported in the literature (0.7–2.7) [48, 50]. No *R* values were determined for the 2‐naphthyl system due to its distinct structural behavior.

Comparison of the phenyl and 1‐naphthyl systems shows nearly constant *d*_1_ but a reduced *d*_2_, indicating flatter ladder structures (Scheme 2d). No significant differences are observed between triol‐ and dimer‐derived materials, suggesting similar architectures. The phenyl system exhibits high ladder regularity with *R* up to 1.91, exceeding literature values of 1.15 [48], whereas the 1‐naphthyl system shows pronounced precursor dependence: silanetriol‐derived materials are more regular (*R* = 1.96) than those from dimers (*R*  =  1.50), reflecting increased defect density. This is attributed to the lower mobility of dimeric precursors, particularly in the sterically demanding 1‐naphthyl system.

The 2‐naphthyl system differs fundamentally (Figure 7). Instead of broad ladder reflections, two sharp reflections at approximately 5 and 15° 2*θ* with a 3:1 spacing ratio are observed, indicating preferred orientation. Comparable diffraction patterns have been associated with helix‐like ladder structures composed of hexagonally packed rods [45]. Powder XRD, measured in glass capillaries (Figure S23), yields nearly identical patterns without additional reflections, supporting anisotropic morphologies. The only change was the intensity ratio of the two reflections, which increased from *I*_1_/*I*_2_ = 0.14 to 0.27, providing further evidence for preferred orientation, consistent with anisotropic morphologies such as rods or platelets. A platelet‐like structure is favored over rod‐like arrangements due to the limited number of reflections and consistency with layered crystal structures, suggesting largely platelets with defined interlayer spacing.

FT‐IR data support the presence of ring‐like units, which may assemble into extended sheet‐like structures. SEM analysis (Figure S24) further indicates platelet morphology. Fluorescence spectroscopy corroborates increasing aryl intercalation, as strong solid‐state excimer emission is observed for both naphthyl systems.

A comparison with previously reported melting gels [49] reveals significantly higher *R* values for the systems investigated here, indicating a higher degree of structural order. The largest discrepancy is observed for the 2‐naphthyl system: while literature reports show behavior similar to phenyl and 1‐naphthyl analogues, with two broad reflections assigned to *d*_1_ and *d*_2_ distances, the present study reveals pronounced preferred orientation, indicative of amorphous yet layered structures.

These results demonstrate that materials obtained via classical sol–gel synthesis (wet‐chemical) [49] differ fundamentally from those produced by solid‐state thermal condensation.

### 2.6 Thermal Analysis

#### 2.6.1 Thermal Cleavage

Condensed organosilsesquioxanes generally exhibit high thermal stability above 400 °C. However, previous studies [38] have shown that organic substituents can be cleaved from the siloxane network at elevated temperatures via substitution rather than homolytic bond cleavage [61]. While earlier work focused on monosilanols and silanediols, the present study examines silanetriols, assuming comparable aryl cleavage behavior for the corresponding dimers.

Experiments followed an established protocol (Figure S25): [38] silanetriols (150–200 mg) were heated at 200 °C for 4 h, and volatile products were transferred under argon into acetone‐d_6_ for NMR analysis, using vinyltrimethoxysilane as an internal standard.

No aryl cleavage was detected for the phenyl and 1‐naphthyl systems; only water from condensation reactions was observed (Ph: 98.73%; 1‐Np: 74.51%; mol% per silanetriol; Figures S26, S27). In contrast, 2NpTriol produced trace amounts of naphthalene (0.68%) alongside water (70.16%), confirmed by NMR and visual observation of crystalline naphthalene (Figure S28). The significantly lower naphthalene yield compared to naphthylsilanediols (up to 15%) [38] indicates reduced cleavage tendency, consistent with reports that removal of the final organic substituent is unfavorable [38].

This reduced reactivity is attributed to increased cross‐linking within the silsesquioxane network, limiting access to silicon centers. Additionally, coordination by three oxygen atoms enhances silicon nucleophilicity, potentially suppressing substitution by neutral or hydroxide species.

#### 2.6.2 DSC Measurements

DSC analysis was used to determine the melting behavior of the silanols, which is relevant for condensation since increased molecular mobility above the melting point accelerates the process. Additionally, DSC provides access to glass transition temperatures arising from partial condensation.

**FIGURE 8:**
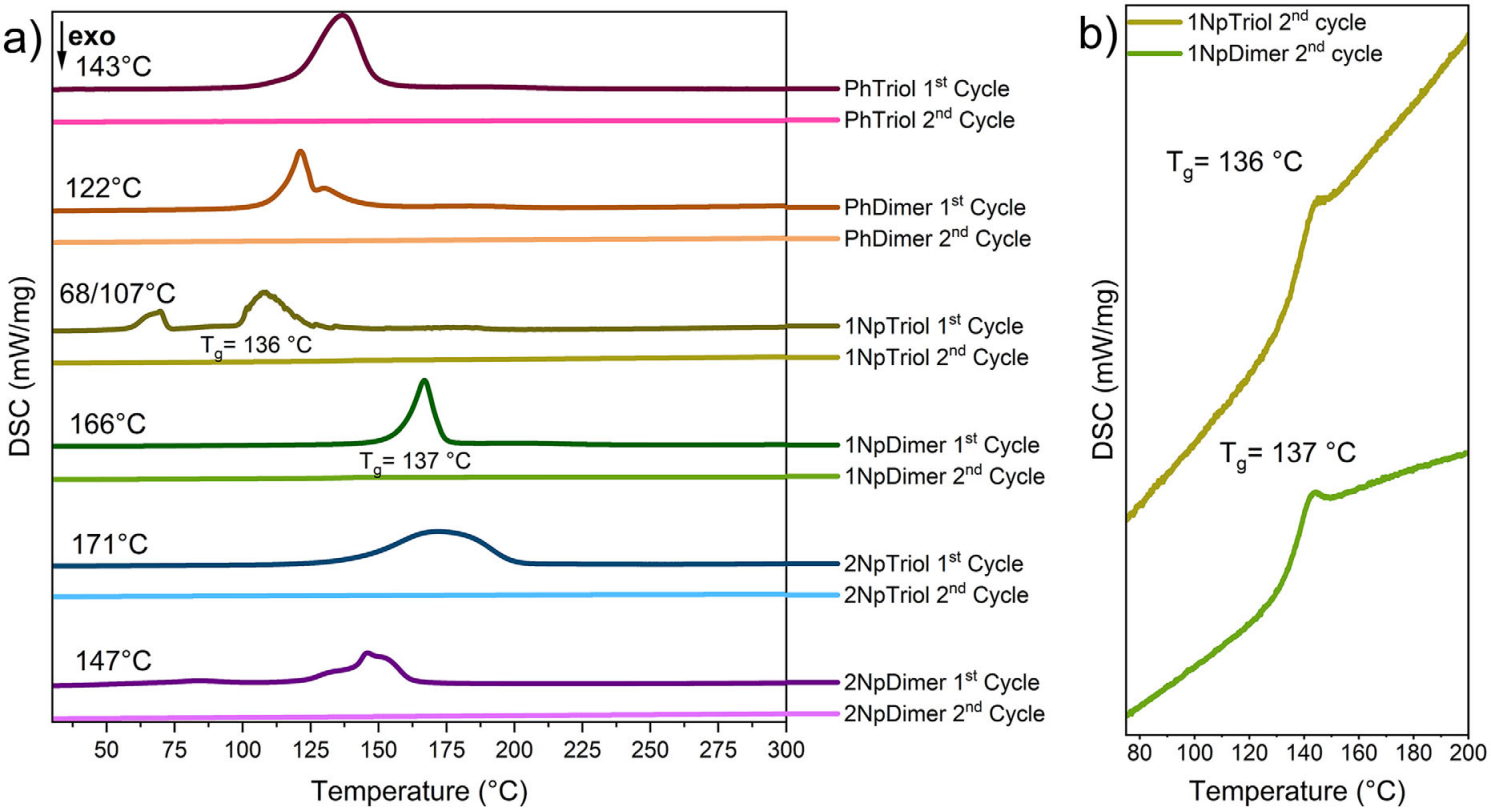
DSC curves of all investigated compounds (a) and enlarged representation of the glass transition temperatures of the 1‐naphthyl system (b).

In the first heating cycle, all silanols show a single endothermic signal, except for 1NpTriol (Figure 8). Minor shoulders are observed for PhDimer and 2NpDimer. The sharp peaks of the phenyl system and 1NpDimer indicate melting, followed by rapid condensation due to increased mobility. The shoulders are attributed to condensation occurring during melting, accompanied by transient species formation and water evaporation.

For 1NpTriol, two endothermic peaks are observed: the first at 68 °C corresponds to solid‐state condensation, as confirmed by ^1^H NMR (Figure S29), and the second at 107 °C to water evaporation, although partial evaporation occurs at lower temperatures, as indicated by TG data. Condensation is complete after the first heating cycle, and no melting transitions are observed in the second cycle. The 2‐naphthyl system exhibits significantly broader endothermic features, indicating ongoing condensation rather than a defined melting process, consistent with the absence of visible changes during heating.

In the second heating cycle, nearly identical glass transition temperatures are observed for the 1‐naphthyl system (136 and 137 °C for triol and dimer). No glass transitions are detected for the phenyl or 2‐naphthyl systems, correlating with their insolubility. In contrast, the partial mobility of 1‐naphthyl condensates enables segmental motion and thus observable T_g_ values. This is consistent with literature reports for phenyl systems [48].

#### 2.6.3 Thermo‐Gravimetric Analysis

**FIGURE 9:**
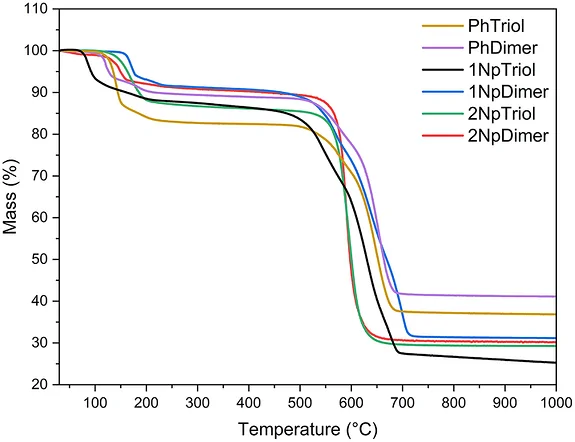
Results of thermogravimetric analyses of the discussed compounds.

Thermogravimetric analysis shows similar behavior for all compounds, with two distinct mass‐loss steps (Figure 9). The first corresponds to condensation with water release (8–17%), while the second reflects Si─C bond cleavage and loss of aryl groups (45–58%) (Table 2). The lower relative water loss of dimers arises from their higher molecular weight, whereas the molar water loss is higher due to the greater number of silanol groups.

**TABLE 2:** Overview of the results of the thermogravimetric analysis of all silanols.

Compound	PhTriol	PhDimer	1NpTriol	1NpDimer	2NpTriol	2NpDimer
**1^st^ Mass loss (%)**	16.76	9.92	11.93	8.32	13.13	7.42
**1 ^st^ Mass loss (°C)**	102	98	67	149	118	116
**Water loss (mol%)**	145.45	162.25	136.71	182.36	150.46	162.63
**2^nd^ Mass loss (%)**	44.65	47.15	58.77	58.35	55.57	58.75
**2 ^nd^ Mass loss (°C)**	496	506	456	466	522	514
**Organics (C, H)**	51.29	53.75	63.12	65.49	63.12	65.49
**Residual mass (%)**	36.78	41.20	25.45	31.26	29.19	30.28
**Residual mass SiO_2_ calculated (%)**	38.47	40.82	29.13	30.46	29.13	30.46

A key difference is observed in the onset of the first mass‐loss step (Table 2): for 1NpTriol, condensation begins around 70 °C, while for the corresponding dimer it starts around 150 °C. This pronounced shift is unique to the 1‐naphthyl system; other systems show similar onset temperatures comparing the corresponding triols and dimers, consistent with DSC data (Figure 8). This behavior reflects the high condensation tendency of the 1‐naphthyl triol, which also complicates its isolation.

The second mass‐loss step occurs between 456 °C and 522 °C, following the order 1‐naphthyl < Ph < 2‐naphthyl. Mass losses of dimers are comparable (1‐naphthyl) or higher (Ph, 2‐naphthyl) than those of the corresponding triols.

Comparison with the total organic content (C, H) shows that the second‐stage mass loss is lower than expected, due to partial hydrogen loss during condensation, incomplete mass loss, and minor volatilization across the temperature range.

Residual masses at 1000 °C agree well with the calculated SiO_2_ mass for the 2‐naphthyl system. For phenyl and 1‐naphthyl systems, triols show lower and dimers higher residual masses than expected. Higher dimer residues indicate incomplete oxidation with remaining Si─C bonds, whereas lower triol residues suggest loss of silicon‐containing species prior to full oxidation. Overall, the results are consistent with related literature systems [48, 49].

### 2.7 Molecular Weight

**FIGURE 10:**
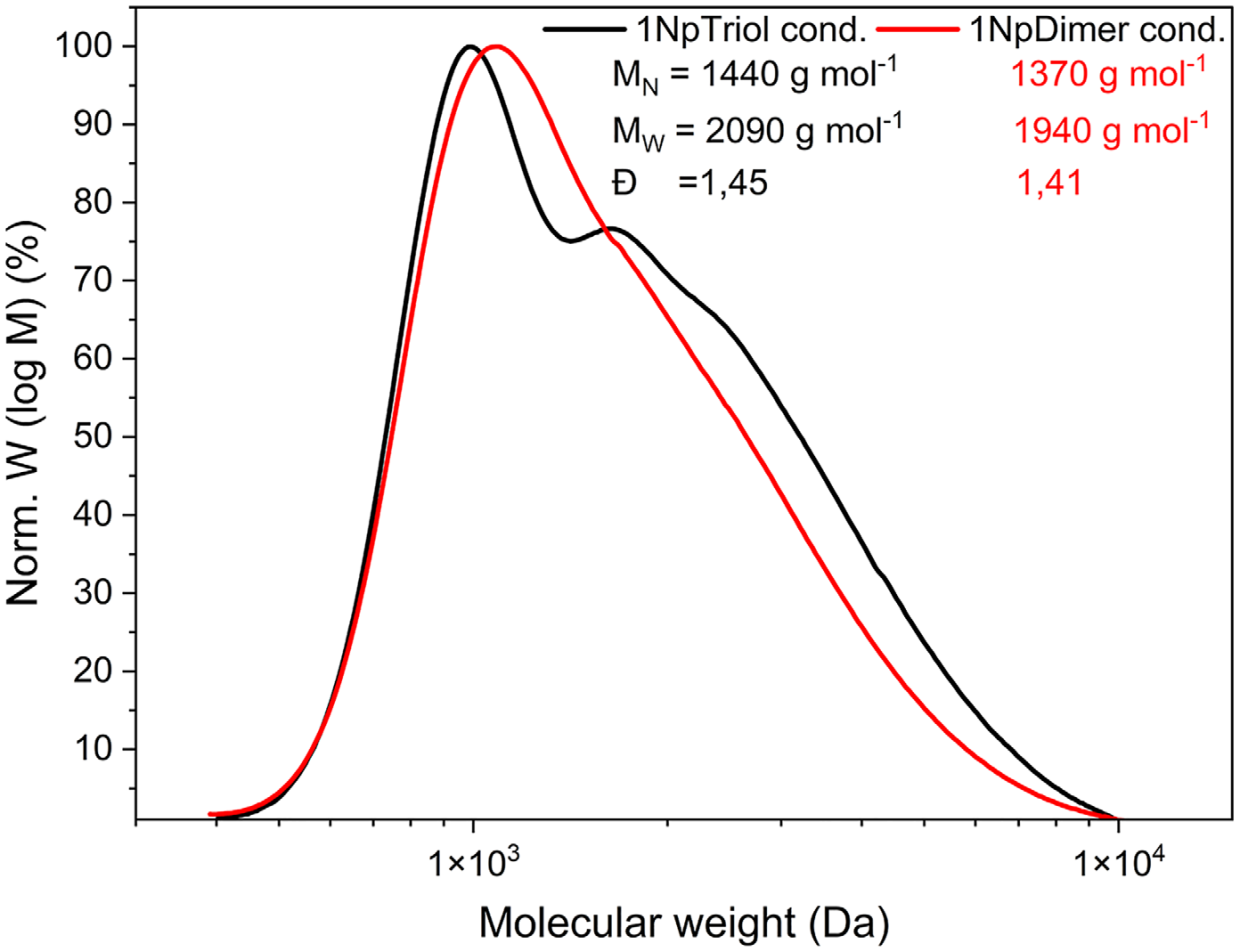
SEC (RI detector) measurements of the condensed 1Np system.

Since only the 1Np system remained soluble after condensation, it was the only one that could be analyzed by SEC and FT‐ICR MS. SEC measurements (THF, polystyrene standards) reveal very similar molecular weight distributions for condensates derived from 1NpTriol and 1NpDimer, with a slight high‐mass shoulder for the triol‐derived material (Figure 10). The weight‐average molar masses (M_W_) lie between 1900 and 2200 g mol^−1^, corresponding to average chain lengths of approximately 8–11 repeat units. Slightly lower molar masses are observed for the dimer‐derived species, likely due to reduced precursor mobility and increased steric demand.

Compared to trialkoxysilane‐derived oligosilsesquioxanes, these values are slightly higher [48, 49], whereas thermally condensed silanediols yield significantly shorter chains [38], consistent with the higher condensation tendency of silanetriols. Polydispersity indices (1.4–1.5) are in good agreement with literature values [48, 49]. Overall, both precursors yield very similar condensation products. Absolute molar masses should be interpreted cautiously, as polystyrene standards better represent linear polymers than partially cross‐linked silsesquioxanes.

MALDI‐ToF spectra (Figure S30) show alternating mass differences of 170 and 188 Da for both products, consistent with stepwise addition of one repeat unit accompanied by the loss of one or two water molecules. No additional regular patterns or signals indicative of intramolecular condensation were observed.

## 3 Conclusions

The structure and thermal condensation behavior of phenyl‐, 1‐naphthyl‐, and 2‐naphthyl‐substituted silanetriols and their corresponding disiloxanetetraols were systematically investigated to assess their differences from conventional trialkoxysilanes and the resulting material structures. Solution NMR confirmed successful synthesis and strong aryl‐induced deshielding, while solid‐state NMR revealed predominantly T^2^ and T^3^ units and a higher degree of condensation than in alkoxysilane‐derived systems. This reflects a fundamental difference in reactivity, as silanols undergo direct, hydrogen‐bond‐mediated condensation without alcohol formation.

Solid precursors show structures with characteristic interlayer distances, confirmed by PXRD, that change upon condensation and correlate with excimer formation observed by fluorescence spectroscopy. The latter reveals strong intra‐ and intermolecular excimer emission in condensed materials, while only dimeric precursors show excimer formation in solution, indicating preorganization at the molecular level.

FT‐IR analyses show ladder‐type structures for the condensed phenyl and 1‐naphthyl systems, whereas the 2‐naphthyl system forms distinct cyclic motifs. The PXRDs of the condensed phenyl and 1‐naphthyl systems confirm the ladder‐like structures, whereas the 2‐naphthyl system shows sharp reflections and pronounced preferred orientation, consistent with a platelet‐like, partially ordered morphology. These structures differ fundamentally from classical sol‐gel‐derived melting gels.

Thermal analysis indicates condensation with water release at 67–149 °C and aryl decomposition at 456–522 °C, with only minor aryl cleavage observed. DSC shows melting‐induced condensation for most systems and glass transition behavior exclusively for the 1‐naphthyl system. SEC analysis reveals low‐molecular‐weight oligomers with slightly higher molar masses than comparable alkoxysilane‐derived materials.

Overall, silanetriols and disiloxanetetraols enable direct condensation pathways that lead to highly ordered, structurally distinct materials. While precursor type mainly affects kinetics and defect density, the resulting network topology is governed by the aryl substituent. Consequently, silanol‐based systems differ fundamentally from alkoxysilane‐derived materials and cannot be classified as conventional melting gels.

## Conflicts of Interest

The authors declare no conflicts of interest.

## Use of Generative AI and AI‐Assisted Technologies in the Writing Process

During the preparation of this work, the authors utilized AI tools, including Deepl.com and ChatGPT to enhance the clarity of the language and to correct spelling, punctuation, and grammatical errors. After using these tools, the authors carefully reviewed and edited the content as necessary and take full responsibility for the accuracy and integrity of the publication.

## Supporting information



The authors have cited additional references within the Supporting Information [62, 63, 64, 65].

## Data Availability

The data that support the findings of this study are available in the supplementary material of this article.
