# Supplementary material for: Substituent‐Dependent Structural Trends in Thermal Condensation of Silanetriols and Disiloxanetetraols
Source: Chemistry. 2026 Jun 12;32(36):e71242. doi: 10.1002/chem.71242 (PMC13602886; doi:10.1002/chem.71242)
Supplement: Supplementary file 1 [file CHEM-32-e71242-s001.docx]

**Substituent-Dependent Structural Trends in Thermal Condensation of Silanetriols and Disiloxanetetraols**

Jan-Falk Kannengießer[a], Dr. Svenja Pohl [a] Dr. Bernd Morgenstern[a], Dr. Oliver Janka[a], and Prof. Dr. Guido Kickelbick[a]*

[a]	Saarland University, Inorganic Solid-State Chemistry, Campus, Building C4 1, 66123 Saarbrücken, Germany

* Corresponding author: E-Mail [guido.kickelbick@uni-saarland.de](mailto:guido.kickelbick@uni-saarland.de)

**Supporting Information**

**Experimental** …………………………………………………………………………………………………………………………………….4

**Figure S1.** ^1^H NMR spectrum of phenylsilanetriol (PhTriol) measured in DMSO-d_6_.	7

**Figure S2.** ^1^H NMR spectrum of 1,3-diphenyldisiloxane-1,1,3,3-tetraol (PhDimer) measured in DMSO-d_6_.	7

**Figure S3.** ^1^H NMR spectrum of 1-naphthylsilanetriol (1NpTriol) measured in DMSO-d_6_.	8

**Figure S4.** ^1^H NMR spectrum of 1,3-di(naphthalen-1-yl)disiloxane-1,1,3,3-tetraol (1NpDimer) measured in DMSO-d_6_.	8

**Figure S5.** ^1^H NMR spectrum of 2-naphthylsilanetriol (2NpTriol) measured in DMSO-d_6_.	9

**Figure S6.** ^1^H NMR spectrum of 1,3-di(naphthalen-2-yl)disiloxane-1,1,3,3-tetraol (2NpDimer) measured in DMSO-d_6_.	9

**Figure S7.** ^29^Si NMR spectrum of phenylsilanetriol (PhTriol) measured in DMSO-d_6_.	10

**Figure S8.** ^29^Si NMR spectrum of 1,3-diphenyldisiloxane-1,1,3,3-tetraol (PhDimer) measured in DMSO‑d_6_.	10

**Figure S9.** ^29^Si NMR spectrum of 1-naphthylsilanetriol (1NpTriol) measured in DMSO-d_6_.	11

**Figure S10.** ^29^Si NMR spectrum of 1,3-di(naphthalen-1-yl)disiloxane-1,1,3,3-tetraol (1NpDimer) measured in DMSO-d_6_.	11

**Figure S11.** ^29^Si NMR spectrum of 2-naphthylsilanetriol (2NpTriol) measured in DMSO-d_6_.	12

**Figure S12.** ^29^Si NMR spectrum of 1,3-di(naphthalen-2-yl)disiloxane-1,1,3,3-tetraol (2NpDimer) measured in DMSO-d_6_.	12

**Figure S13.** FT-IR spectra of phenylsilanetriol (PhTriol), 1,3-diphenyldisiloxane-1,1,3,3-tetraol (PhDimer) and their condensed products.	13

**Figure S14.** FT-IR spectra of 1-naphthylsilanetriol (1NpTriol), 1,3-di(naphthalen-1-yl)disiloxane-1,1,3,3-tetraol (1NpDimer) and their condensed products.	13

**Figure S15.** FT-IR spectra of 2-naphthylsilanetriol (2NpTriol), 1,3-di(naphthalen-2-yl)disiloxane-1,1,3,3-tetraol (2NpDimer) and their condensed products.	14

**Figure S16.** Fluorescence spectrum of solid 1-naphthylsilanetriol (1NpTriol) and the corresponding dilution series.	14

**Figure S17.** Fluorescence spectrum of solid 1,3-di(naphthalen-1-yl)disiloxane-1,1,3,3-tetraol (1NpDimer) and the corresponding dilution series.	15

**Figure S18.** Fluorescence spectrum of solid 2-naphthylsilanetriol (2NpTriol) and the corresponding dilution series.	15

**Figure S19.** Fluorescence spectrum of solid 1,3-di(naphthalen-2-yl)disiloxane-1,1,3,3-tetraol (2NpDimer) and the corresponding dilution series.	16

**Figure S20.** Fluorescence spectrum of the solid condensed 1-naphthylsilanetriol (1NpTriol cond.) and the corresponding dilution series.	16

**Figure S21.** Fluorescence spectrum of the solid condensed 1,3-di(naphthalen-1-yl)disiloxane-1,1,3,3-tetraol (1NpDimer cond.) and the corresponding dilution series.	17

**Figure S22.** Fluorescence spectrum of the solid condensed 1-naphthylsilanetriol (1NpTriol cond.), 1,3-di(naphthalen-1-yl)disiloxane-1,1,3,3-tetraol (1NpDimer cond.), 2-naphthylsilanetriol (2NpTriol cond.) and 1,3-di(naphthalen-2-yl)disiloxane-1,1,3,3-tetraol (2NpDimer cond.).	17

**Figure S23.** PXRD measurements measured with flat sample holder and capillary of the condensed 2‑naphthylsilanetriol (2NpTriol cond.) and 1,3-di(naphthalen-2-yl)disiloxane-1,1,3,3-tetraol (2NpDimer cond.).	19

**Figure S24.** SEM image of the product from the thermal condensation of 2NpTriol. Magnification: 2000x (Polaroid 545); EHT: 5.00 kV; WD: 6.8 mm.	20

**Figure S25.** Schematic diagram of the setup of NMR distillation for the investigation of by-products formed during condensation.	20

**Figure S26.** ^1^H NMR spectrum of the volatile products from NMR distillation of phenylsilanetriol (PhTriol). Measured in acetone-d_6_ with the addition of 9.8 mg of vinyltrimethoxysilane.	21

**Figure S27.** ^1^H NMR spectrum of the volatile products from NMR distillation of 1-naphthylsilanetriol (1NpTriol). Measured in acetone-d_6_ with the addition of 8.9 mg of vinyltrimethoxysilane.	21

**Figure S28.** ^1^H NMR spectrum of the volatile products from NMR distillation of 2-naphthylsilanetriol (2NpTriol). Measured in acetone-d_6_ with the addition of 9.9 mg of vinyltrimethoxysilane.	22

**Figure S29.** ^1^H NMR spectrum of 1-naphthylsilanetriol (1NpTriol) after heating up to 80°C, to investigate the first peak in DSC.	22

**Figure S30.** FT-ICR MS (a) measurements of the condensed 1Np system. Enlarged FT-ICR MS section from the 1Np system (b).	23

**Figure S31.** SEC (THF as eluent) measurements of condensed 1-naphthylsilanetriol (1NpTriol) (a) and 1,3-di(naphthalen-1-yl)disiloxane-1,1,3,3-tetraol (1NpDimer) (b).	24

**Experimental**

Materials

Toluene (for synthesis), acetic acid (99.8%), methanol (98% for synthesis) was purchased from the central chemical storage of Saarland University. Phenyltrimethoxysilane (97%) was purchased from ABCR GmbH (Karlsruhe, Germany). Hydrochloric acid (37%) was purchased from VWR Chemicals (Darmstadt, Germany). All NMR spectra were recorded in DMSO-d6 (99.8%, Deutero GmbH, Kastellaun, Germany).

All chemicals used were used without further purification. The reactions were not carried out under an inert atmosphere.

1-Naphthyltrimethoxysilane and 2-naphthyltrimethoxysilane were synthesized by Grignard reactions of the corresponding bromonaphthalenes with tetramethoxysilane. The synthesis and purification were carried out as in previous work.^[1-2]^

Characterization methods

Solution NMR spectra were obtained using a Avance III 400 MHz spectrometer (Bruker BioSpin, Rheinstetten, Germany) at 400.13 MHz for ^1^H NMR and 79.49 MHz for ^29^Si NMR. Further NMRs were recorded using a Bruker Avance III 300 spectrometer (Bruker BioSpin, Rheinstetten, Germany) at 300 MHz for ^1^H NMR and 59.60 MHz for ^29^Si NMR.

Solid-state SPE-MAS NMR spectra were recorded on an Avance III 400WB spectrometer (Bruker Biospin, Rheinstetten, Germany) using 4 mm inner diameter ZrO_2_ rotors with 13 kHz rotation frequency. The resonance frequencies were 79.53 MHz for ^29^Si NMR spectra. The spectra were analyzed and integrated with MestReNova^[3]^ using a generalized-Lorentzian function.

ATR-FTIR spectra were measured on a Vertex 70 spectrometer (Bruker Optics, Ettlingen, USA) in attenuated total reflectance mode at 4500-400 cm^−1^ with 4 cm^−1^ increment and 16 averaged scans.

Single-crystal X-ray diffraction (SCXRD): The data set was collected using a D8 Venture diffractometer (Bruker AXS, Karlsruhe, Germany) with a microfocus sealed tube and a Photon II detector. Monochromated MoK_α_ radiation (λ = 0.71073 Å) was used. Data were collected at 133(2) K and corrected for absorption effects using the multi-scan method. The structure was solved by direct methods using SHELXT^[4]^ and was refined by full matrix least squares calculations on F2 (SHELXL2018)^[5]^ in the graphical user interface Shelxle^[6]^. All non-H-atoms were located on the electron density maps and refined anisotropically. C-bound hydrogen atoms were placed in positions of optimized geometry and treated as riding atoms. Their isotropic displacement parameters were coupled to the corresponding carrier atoms by a factor of 1.2 (CH). The positional parameters of the O-bonded hydrogen atoms H1O and H2O were refined using isotropic displacement parameters which were set at 1.5 times the *U_eq_* value of the parent atom. Restraints of 84(1) pm were used for the O∙∙∙H bond lengths. Deposition Number 2542304 contain the supplementary crystallographic data for this paper. These data are provided free of charge by the joint Cambridge Crystallographic Data Centre and Fachinformationszentrum Karlsruhe <url href="http://www.ccdc.cam.ac.uk/structures">Access Structures service</url>.

Powder X-ray diffraction (PXRD) patterns of the pulverized samples were recorded at room temperature on a D8-A25-Advance diffractometer (Bruker AXS, Karlsruhe, Germany) in Bragg-Brentano θ-θ-geometry (goniometer radius 280 mm) with non-monochromatic Cu *K*_α1,2_-radiation (*λ*_1_ = 154.0596, *λ*_2_ = 154.4426 pm). A 12 µm Ni foil working as *K_β_* filter and a variable divergence slit were mounted at the primary beam side. A LYNXEYE detector with 192 channels and an opening angle of 1° alongside a motorized anti-scatter slit was used at the secondary beam side. Experiments were carried out in a 2θ range of 3 to 60° with a step size of 0.013° and a total scan time of 1h. For the capillary measurements, the powder was filled in glass capillaries with 1 mm outer diameter. The capillaries were mounted on a goniometer using putty and centered in the beam. Measurements were conducted with the same geometry described above, but on the primary beam side the variable divergence slit was replaced by a Göbel mirror. The measurements were also conducted between 3 and 60° 2θ with the capillary rotating at 25 rpm.

Differential scanning calorimetry (DSC) was carried out on a DSC 204 F1 Phoenix (Netzsch, Selb, Germany). Measurements were performed in nitrogen atmosphere (40 + 60 mL min^−1^) and a heating of 10 K min^−1^ and cooling rate of 15 K min^−1^ was used. The samples were measured in perforated aluminum crucibles.

Thermo gravimetric analysis (TGA) measurements were performed using a PERSEUS TG 209 F1 Libra (NETZSCH-Gerätebau GmbH, Selb, Germany and Bruker Optics, Ettlingen, Germany). The measurements were carried out at a 10 K min^−1^ heating rate in the temperature range between 25 and 1000 °C under synthetic air flow (40 mL min^−1^, N_2_/O_2_ 75:25%).

Fluorescence spectroscopy was performed applying a FluoroMax 4 spectrofluorometer (Horiba Scientific, Kyoto, Japan) with an excitation wavelength of 285 nm. The samples were measured as solids and as solution in dimethylsulfoxide.

The C, H, and N content of the compounds was determined using a Vario Micro cube (Elementar Analysensysteme GmbH, Langenselbold, Germany).

Size exclusion chromatography (SEC) was performed with a PSS SECcurity2 system composed of a 1260 IsoPump-G7110B (Agilent Technologies, Santa Clara, CA, USA) and a 1260 VW-detector G7162A at 270 nm (Agilent Technologies) at 25 °C, with THF as the mobile phase (flow rate 1 mL min^−1^) on a SDV column set (SDV 10^3^, SDV 10^5^, SDV 10^6^) from PSS (Polymer Standard Service, Mainz, Germany). Calibration was carried out using PS standards (from PSS).

Matrix-assisted laser desorption/ionization time of flight (MALDI-ToF) spectra were recorded
with a Bruker Daltonik Autoflex speed TOF/TOF with a positive reflection mode. The analyzer was a time-of-flight mass spectrometer. For the matrix the nonpolar, aprotic 2-[(2E)-3-(4-tert-butylphenyl)-2-methylprop-2-enylidene]-malononitrile (DCTB) were used. Calibration was carried out using PEG standards with sodium salt addition.

The Scanning electron microscope (SEM) picture were taken with a Zeiss SIGMA VP with a Schottky field emitter and a secondary electron detector. An Extra High Tension (EHT) of 5 kV was used with a working distance of 6.8 mm. The picture was taken with a magnification of 2000x (Polaroid 545).

Synthetic procedures

**Phenylsilanetriol (PhTriol)**: Synthesis based on literature.^[7-8]^ To a 25 mL round flask with stirrer, 10 g (50.4 mmol) of phenyltrimethoxysilane, 5.56 mL of distilled water and 0.026 mL of acetic acid (99%) were added. The reaction mixture was stirred at room temperature for 3 hours. The white solid was filtered and washed with toluene. The product (white solid) was dried under high vacuum. A yield of 49.95% was obtained.

^1^H NMR (400 MHz, DMSO-d_6_): δ = 7.61-7.58 ppm (m, 2H); 7.35-7.28 ppm (m, 3H); 6.39 ppm (s, 3H).

^29^Si NMR (80 MHz, DMSO-d_6_): δ = −54.83 ppm.

Elemental analysis: calculated [%]: C, 46.13; H, 5.16. Found [%]: C, 46.29; H, 4.95.

**1,1,3,3-Tetramethoxy-1,3-diphenylsiloxane**: Synthesis based on literature.^[9]^ 79.33 g Phenyltrimethoxsilane (400 mmol) and 20 ml methanol were placed in a 200 ml flask. A mixture of 2.7 ml water (0.15 mol), 0.3 mL HCl (1M) and 20 ml methanol was added over 30 minutes at room temperature. After a further 24 hours of stirring at room temperature, the mixture was distilled under high vacuum (2.0 x 10-2 mbar, 110-120 °C). The result was a colorless oil (30.97 g, 44%).

^1^H NMR (400 MHz, acetone-d_6_): δ = 7.70-7.68 ppm (m, 4H); 7.49-7.40 ppm (m, 6H); 3.60 ppm (s, 12H).

^29^Si NMR (80 MHz, acetone-d_6_): δ = −62.97 ppm.

Elemental analysis: calculated [%]: C, 54.83; H, 6.33. Found [%]: C, 53.40; H, 5.62.

**1,3-Diphenyldisiloxane-1,1,3,3-tetraol (PhDimer)**: Synthesis based on previous work.^[1]^ To a snap cap glass with stirrer, 0.5 g of 1,1,3,3-tetramethoxy-1,3-diphenyldisiloxane (1.4 mmol), 0.3 mL of distilled water and 0.012 mL of acetic acid (50%) were added. The reaction mixture was stirred at room temperature for 24 h. The water was removed under high vacuum and the residue obtained was washed several times with toluene. The product (white solid) was dried under high vacuum. A yield of 31.23% was obtained.

^1^H NMR (400 MHz, DMSO-d_6_): δ = 7.66-7.64 ppm (m, 4H); 7.37-7.30 ppm (m, 6H); 6.64 ppm (s, 4H).

^29^Si NMR (80 MHz, DMSO-d_6_): δ = −63.67 ppm.

Elemental analysis: calculated [%]: C, 48.96; H, 4.79. Found [%]: C, 48.79; H, 4.67.

**Naphthalen-1-ylsilanetriol (1NpTriol)**: Synthesis based on previous work.^[1]^ To a snap cap glass with stirrer, 0.5 g of trimethoxy(naphthalen-1-yl)silane (2.01 mmol), 2.0 mL of distilled water and 0.004 mL of acetic acid (10%) were added and cooled in an ice bath. The reaction mixture was stirred for exactly 24 h, without continuous maintenance of cooling. After stirring, the reaction mixture was filtered through a syringe filter (0.5 µm). The filtrates of two batches were combined in a 25 mL round flask, and the water was removed in a high vacuum with the flask in a water bath to prevent freezing. The product (white solid) gave a yield of 19.32%.

^1^H NMR (400 MHz, DMSO-d_6_): δ = 8.44 ppm (d, J = 9.7 Hz, 1H); 7.94-7.88 ppm (m, 3H); 7.52-7.45 ppm (m, 3H); 6.65 ppm (s, 3H).

^29^Si NMR (80 MHz, DMSO-d_6_): δ = −54.83 ppm.

Elemental analysis: calculated [%]: C, 58.23; H, 4.89. Found [%]: C, 57.83; H, 4.72.

**1,3-Di(naphthalen-1-yl)disiloxane-1,1,3,3-tetraol (1NpDimer)**: Synthesis based on previous work.^[1]^ In these previous studies, it was incorrectly assumed that naphthalene-1-ylsilanetriol was obtained. However, the crystal structure subsequently revealed the correct structure. To a snap cap glass with stirrer, 2.0 mL of distilled water and 0.059 mL of acetic acid (50%) were added and cooled in an ice bath. Then 0.5 g of Trimethoxy(naphthalen-1-yl)silane (2.01 mmol) was added in portions over 30 min and stirred for 2 h in an ice bath. A new ice bath was then prepared and the reaction mixture was stirred for 18 h, without continuous maintenance of cooling. The white precipitate was cold filtered off and washed several times with cold toluene. The product (white solid) was dried under high vacuum. A yield of 75.8% was obtained.

^1^H NMR (400 MHz, DMSO-d_6_): δ = 8.46 ppm (d, J = 8.1 Hz, 2H); 8.02-7.88 ppm (m, 6H); 7.48-7.40 ppm (m, 6H); 6.91 ppm (s, 4H).

^29^Si NMR (80 MHz, DMSO-d_6_): δ = −63.62 ppm.

Elemental analysis: calculated [%]: C, 60.89; H, 4.60. Found [%]: C, 59.74; H, 4.90.

**Naphthalen-2-ylsilanetriol (2NpTriol)**: Synthesis based on previous work.^[1]^ To a snap cap glass with stirrer, 0.5 g of trimethoxy(naphtalen-2-yl)silane (2.01 mmol), 2.0 mL of distilled water and 0.059 mL of acetic acid (50%) were added. The reaction mixture was stirred at room temperature for 24 h. The white solid was filtered and washed with toluene. The product (white solid) was dried under high vacuum. A yield of 78.98% was obtained.

^1^H NMR (400 MHz, DMSO-d_6_): δ = 8.17 ppm (s, 1H); 7.92-7.85 ppm (m, 3H); 7.71 ppm (d, J = 8.1 Hz, 1H); 7.52-7.50 ppm (m, 2H); 6.53 ppm (s, 3H).

^29^Si NMR (80 MHz, DMSO-d_6_): δ = −54.37 ppm.

Elemental analysis: calculated [%]: C, 58.23; H, 4.89. Found [%]: C, 57.70; H, 4.98.

**1,1,3,3-Tetramethoxy-1,3-di(naphthalen-2-yl)disiloxane**: Synthesis based on literature.^[9]^ 19.87 g Trimethoxy(naphthalen-2-yl)silane (80 mmol) and 4 ml methanol were placed in a 50 ml round flask. A mixture of 0.54 ml water (30 mmol), 0.06 mL 1 M HCl and 4 ml methanol was added over 30 minutes at room temperature. After a further 48 hours of stirring at room temperature, the mixture was distilled under high vacuum (2.0 x 10^-2^ mbar, > 200 °C). The result was a colorless to yellowish oil (6.56 g, 36.40%).

^1^H NMR (400 MHz, DMSO-d_6_): δ = 8.27 ppm (s, 2H); 7.91-7.87 ppm (m, 6H); 7.74 ppm (dd, J = 8.2, 1.1 Hz, 2H); 6.91 ppm (s, 4H).

^29^Si NMR (80 MHz, DMSO-d_6_): δ = −62.96 ppm.

Elemental analysis: calculated [%]: C, 63.97; H, 5.82. Found [%]: C, 63.11; H, 5.11.

**1,3-di(naphthalen-2-yl)disiloxane-1,1,3,3-tetraol (2NpDimer)**: Synthesis based on previous work.^[1]^ To a snap cap glass with stirrer, 0.5 g of 1,1,3,3-tetramethoxy-1,3-di(naphthalen-2-yl)disiloxane (1.11 mmol), 2.0 mL of distilled water and 0.5 mL of acetic acid (99%) were added. The reaction mixture was stirred at room temperature for 48 h. The white solid was filtered and washed with toluene. The product (white solid) was dried under high vacuum. A yield of 35.42% was obtained.

^1^H NMR (400 MHz, DMSO-d_6_): δ = 8.21 ppm (s, 2H); 7.89-7.76 ppm (m, 8H); 7.54-7.47 ppm (m, 4H); 6.84 ppm (s, 4H).

^29^Si NMR (80 MHz, DMSO-d_6_): δ = −63.68 ppm.

Elemental analysis: calculated [%]: C, 60.89; H, 4.60. Found [%]: C, 58.31; H, 4.53.

**Figure S1.** ^1^H NMR spectrum of phenylsilanetriol (PhTriol) measured in DMSO-d_6_.

**Figure S2.** ^1^H NMR spectrum of 1,3-diphenyldisiloxane-1,1,3,3-tetraol (PhDimer) measured in DMSO-d_6_.

**Figure S3.** ^1^H NMR spectrum of 1-naphthylsilanetriol (1NpTriol) measured in DMSO-d_6_.

**Figure S4.** ^1^H NMR spectrum of 1,3-di(naphthalen-1-yl)disiloxane-1,1,3,3-tetraol (1NpDimer) measured in DMSO-d_6_.

**Figure S5.** ^1^H NMR spectrum of 2-naphthylsilanetriol (2NpTriol) measured in DMSO-d_6_.

**Figure S6.** ^1^H NMR spectrum of 1,3-di(naphthalen-2-yl)disiloxane-1,1,3,3-tetraol (2NpDimer) measured in DMSO-d_6_.

**Figure S7.** ^29^Si NMR spectrum of phenylsilanetriol (PhTriol) measured in DMSO-d_6_.

**Figure S8.** ^29^Si NMR spectrum of 1,3-diphenyldisiloxane-1,1,3,3-tetraol (PhDimer) measured in DMSO‑d_6_.

**Figure S9.** ^29^Si NMR spectrum of 1-naphthylsilanetriol (1NpTriol) measured in DMSO-d_6_.

**Figure S10.** ^29^Si NMR spectrum of 1,3-di(naphthalen-1-yl)disiloxane-1,1,3,3-tetraol (1NpDimer) measured in DMSO-d_6_.

**Figure S11.** ^29^Si NMR spectrum of 2-naphthylsilanetriol (2NpTriol) measured in DMSO-d_6_.

**Figure S12.** ^29^Si NMR spectrum of 1,3-di(naphthalen-2-yl)disiloxane-1,1,3,3-tetraol (2NpDimer) measured in DMSO-d_6_.

**Figure S13.** FT-IR spectra of phenylsilanetriol (PhTriol), 1,3-diphenyldisiloxane-1,1,3,3-tetraol (PhDimer) and their condensed products.

**Figure S14.** FT-IR spectra of 1-naphthylsilanetriol (1NpTriol), 1,3-di(naphthalen-1-yl)disiloxane-1,1,3,3-tetraol (1NpDimer) and their condensed products.

**Figure S15.** FT-IR spectra of 2-naphthylsilanetriol (2NpTriol), 1,3-di(naphthalen-2-yl)disiloxane-1,1,3,3-tetraol (2NpDimer) and their condensed products.

**Figure S16.** Fluorescence spectrum of solid 1-naphthylsilanetriol (1NpTriol) and the corresponding dilution series.

**Figure S17.** Fluorescence spectrum of solid 1,3-di(naphthalen-1-yl)disiloxane-1,1,3,3-tetraol (1NpDimer) and the corresponding dilution series.

**Figure S18.** Fluorescence spectrum of solid 2-naphthylsilanetriol (2NpTriol) and the corresponding dilution series.

**Figure S19.** Fluorescence spectrum of solid 1,3-di(naphthalen-2-yl)disiloxane-1,1,3,3-tetraol (2NpDimer) and the corresponding dilution series.

**Figure S20.** Fluorescence spectrum of the solid condensed 1-naphthylsilanetriol (1NpTriol cond.) and the corresponding dilution series.

**Figure S21.** Fluorescence spectrum of the solid condensed 1,3-di(naphthalen-1-yl)disiloxane-1,1,3,3-tetraol (1NpDimer cond.) and the corresponding dilution series.

**Figure S22.** Fluorescence spectrum of the solid condensed 1-naphthylsilanetriol (1NpTriol cond.), 1,3-di(naphthalen-1-yl)disiloxane-1,1,3,3-tetraol (1NpDimer cond.), 2-naphthylsilanetriol (2NpTriol cond.) and 1,3-di(naphthalen-2-yl)disiloxane-1,1,3,3-tetraol (2NpDimer cond.).

**Table S1.** Crystallographic data and parameter of the structure refinement of 1,3-di(naphthalen-1-yl)disiloxane-1,1,3,3-tetraol.

| Compound | 1,3-Di(naphthalen-1-yl)disiloxane-1,1,3,3-tetraol |
| --- | --- |
| Chemical formula | C_20_H_18_O_5_Si_2_ |
| Molar mass (g/mol) | 394.52 |
| Temperature (K) | 133(2) |
| Wavelength (pm) | 71.073 |
| Crystal system | monoclinic |
| Space group | *P*2_1_*/c* |
| *a* (pm) | 1343.30(8) |
| *b* (pm) | 834.12(4) |
| *c* (pm) | 818.06(5) |
| *β* (°) | 95.789(2) |
| *V* (nm^3^) | 912 |
| *Z* | 2 |
| ρ_calc._ (g/cm^3^) | 1.44 |
| µ (mm^-1^) | 0.2 |
| F(000) | 412 |
| Crystal size (mm) | 0.29 × 0.26 × 0.04 |
| *θ* range (°) | 2.88-27.11 |
| Index range | −17 ≤ *h* ≤ 17  −9 ≤ *k* ≤ 10  −10 ≤ *l* ≤ 10 |
| Total number of reflections | 16915 |
| R_int_, R_σ_ | 0.0243, 0.0462 |
| Independent reflections | 2013 |
| restrictions | 3 |
| Parameters | 130 |
| R_1_, *w*R_2_ (*I* > 2*σ*(*I*)) | 0.0352, 0.0855 |
| R_1_, *w*R_2_ (all data) | 0.0455, 0.0922 |
| S / GoF | 1.066 |
| Highest differential peak e.Å^-3^ | 0.343 |
| Lowest differential hole e.Å^-3^ | −0.330 |
| Refined hydrogen atoms | geometric |

**Figure S23.** PXRD measurements measured with flat sample holder and capillary of the condensed 2‑naphthylsilanetriol (2NpTriol cond.) and 1,3-di(naphthalen-2-yl)disiloxane-1,1,3,3-tetraol (2NpDimer cond.).

**Figure S24.** SEM image of the product from the thermal condensation of 2NpTriol. Magnification: 2000x (Polaroid 545); EHT: 5.00 kV; WD: 6.8 mm.

**Experimental setup of the NMR investigation of aryl cleavage**

**Figure S25.** Schematic diagram of the setup of NMR distillation for the investigation of by-products formed during condensation.

**Figure S26.** ^1^H NMR spectrum of the volatile products from NMR distillation of phenylsilanetriol (PhTriol). Measured in acetone-d_6_ with the addition of 9.8 mg of vinyltrimethoxysilane.

**Figure S27.** ^1^H NMR spectrum of the volatile products from NMR distillation of 1-naphthylsilanetriol (1NpTriol). Measured in acetone-d_6_ with the addition of 8.9 mg of vinyltrimethoxysilane.

**Figure S28.** ^1^H NMR spectrum of the volatile products from NMR distillation of 2-naphthylsilanetriol (2NpTriol). Measured in acetone-d_6_ with the addition of 9.9 mg of vinyltrimethoxysilane.

**Figure S29.** ^1^H NMR spectrum of 1-naphthylsilanetriol (1NpTriol) after heating up to 80°C, to investigate the first peak in DSC.

**Molecular Weight**

The FT-ICR MS spectra show the same oligomers for both condensed species, although the intensity of the corresponding oligomers differs in some cases (Figure S30a). However, this is difficult to understand, since in the case of the 1Np dimer, only masses that are separated from the mass of a dimer should be visible. However, alternating distances of approximately 170 Da and 188 Da are found (Figure S30b). These masses indicate the simple or double condensation of a further 1NpSiO_1.5_ unit, in which one or two water molecules are split off. In the case of the 1NpTriol, these alternating mass increases make perfect sense, as the formation of a ladder structure is presumably visible here. To form a complete ladder structure, another trifunctional unit is first added, resulting in the removal of one water molecule. After that, another unit can be added, but this time it condenses twice, resulting in the removal of two water molecules. In the case of the 1Np dimer, the presence of odd chain lengths indicates a cleavage of the Si-O-Si bond, which makes these chain lengths possible. This does not always have to happen; only a few cleavages would be sufficient to explain several oligomers with odd chain lengths. Such a cleavage would be possible, for example, through backbiting, which is already known to occur in other polysiloxanes. However, no further regularities could be found within the peak groups of an oligomer, which was found in the literature^[10-12]^. These peak groups differ from each other in both condensed species and also change within a spectrum. This indicates that many different types of oligomers are present, for example ladder, ring, or cage structures.

**Figure S30.** FT-ICR MS (a) measurements of the condensed 1Np system. Enlarged FT-ICR MS section from the 1Np system (b).

**Figure S31.** SEC (THF as eluent) measurements of condensed 1-naphthylsilanetriol (1NpTriol) (a) and 1,3-di(naphthalen-1-yl)disiloxane-1,1,3,3-tetraol (1NpDimer) (b).

**References**

[1]	J. F. Kannengiesser, M. Briesenick, D. Meier, V. Huch, B. Morgenstern, G. Kickelbick, *Chem. Eur. J.* **2021**, *27*, 16461-16476.

[2]	M. Briesenick, M. Gallei, G. Kickelbick, *Macromolecules* **2022**, *55*, 4675-4691.

[3]	14.2.0-26256 ed., Mestrelab Research S.L., **2020**.

[4]	G. M. Sheldrick, *Acta Cryst.* **2015**, *A71*, 3-8.

[5]	G. M. Sheldrick, *Acta Cryst.* **2015**, *C71*, 3-8.

[6]	C. B. Hübschle, G. M. Sheldrick, B. Dittrich, *J. Appl. Crystallogr.* **2011**, *44*, 1281-1284.

[7]	S. D. Korkin, M. I. Buzin, E. V. Matukhina, L. N. Zherlitsyna, N. Auner, O. I. Shchegolikhina, *J. Organomet. Chem.* **2003**, *686*, 313-320.

[8]	L. J. Tyler, *J. Am. Chem. Soc.* **1955**, *77*, 770-771.

[9]	Z.-X. Zhang, J. Hao, P. Xie, X. Zhang, C. C. Han, R. Zhang, *Chem. Mater.* **2008**, *20*, 1322-1330.

[10]	S. Pohl, M. Gallei, G. Kickelbick, *Macromolecules* **2025**, *58*, 1298-1313.

[11]	W. E. Wallace, C. M. Guttman, J. M. Antonucci, *Polymer* **2000**, *41*, 2219-2226.

[12]	W. E. Wallace, C. M. Guttman, J. M. Antonucci, *J. Am. Soc. Mass Spectrom.* **1999**, *10*, 224-230.
